# Visibility and transmission: complexities around promoting hand hygiene in young children – a qualitative study

**DOI:** 10.1186/s12889-019-6729-x

**Published:** 2019-04-11

**Authors:** Ruby Biezen, Danilla Grando, Danielle Mazza, Bianca Brijnath

**Affiliations:** 1 Department of General Practice, Monash University, Building 1, 270 Ferntree Gully Road, Notting Hill, VIC 3168 Australia; 2 School of Science, RMIT University, Building 223, Level 1, Bundoora Campus, Plenty Road, Bundoora, VIC 3083 Australia; 3 National Ageing Research Institute LTD, 34-54 Poplar Road, Parkville, VIC 3052 Australia

**Keywords:** Hand hygiene, Hand washing, Transmission, Respiratory tract infection, Children, Primary care providers, Primary care

## Abstract

**Background:**

Effective hand hygiene practice can reduce transmission of diseases such as respiratory tract infections (RTIs) and gastrointestinal infections, especially in young children. While hand hygiene has been widely promoted within Australia, primary care providers’ (PCPs) and parents’ understanding of hand hygiene importance, and their views on hand hygiene in reducing transmission of diseases in the community are unclear. Therefore, the aim of this study was to explore the views of PCPs and parents of young children on their knowledge and practice of hand hygiene in disease transmission.

**Methods:**

Using a cross-sectional qualitative research design, we conducted 30 in-depth interviews with PCPs and five focus groups with parents (*n* = 50) between June 2014 and July 2015 in Melbourne, Australia. Data were thematically analysed.

**Results:**

Participants agreed that hand hygiene practice was important in reducing disease transmissions. However, barriers such as variations of hand hygiene habits, relating visibility to transmission; concerns around young children being obsessed with washing hands; children already being ‘too clean’ and the need to build their immunity through exposure to dirt; and scepticism that hand hygiene practice was achievable in young children, all hindered participants’ motivation to develop good hand hygiene behaviour in young children.

**Conclusion:**

Despite the established benefits of hand hygiene, sustained efforts are needed to ensure its uptake in routine care. To overcome the barriers identified in this study a multifaceted intervention is needed that includes teaching young children good hand hygiene habits, PCPs prompting parents and young children to practice hand hygiene when coming for an RTI consultation, reassuring parents that effective hand hygiene practice will not lead to abnormal psychological behaviour in their children, and community health promotion education campaigns.

## Background

Hand hygiene, including hand washing with soap and water, or the use of hand sanitizers, has been shown to reduce transmission of infectious diseases [1–3], especially gastrointestinal and respiratory tract infections [4]. Young children < 5 years of age are most at risk, in particular those attending childcare or preschool [5–7].

Effective hand hygiene practice in community settings, has demonstrated a reduction of infections occurring in childcare [7–10], schools [2, 11–13], and in the home [14–16]. According to Aiello et. al’s meta-analysis [4] improvements in hand hygiene resulted in a 21% reduction in respiratory illnesses and a 31% reduction in gastrointestinal illnesses in community-based settings. The importance of hand hygiene practice in the prevention of infectious diseases was emphasized in all 30 studies included in this meta-analysis.

Studies from Europe, US, and the UK have also shown that hand hygiene interventions in the community can increase hand hygiene compliance among children [17–19]. For example, interventions involving teacher modelling hand hygiene to school children [20], improving educator’s knowledge and attitude towards hand hygiene [21], and the use of alcohol-based sanitizers [15, 22, 23] have significantly reduced illness absenteeism in schools. However, factors such as lack of time to practice hand hygiene, poor adult modelling of regular hand washing, limited facilities including available sinks, soap and water, and the lack of knowledge regarding the importance of hand hygiene have hindered the compliance and sustainability of good hand hygiene practice [24, 25].

Despite wide promotion of hand hygiene in Australia [26] and good evidence that effective hand hygiene practice reduces infectious disease transmission, to date no studies have measured the efficacy and sustainability of hand hygiene practice in the Australian primary care setting. Thus, it is unclear whether primary care providers (PCPs) and their patients follow recommended protocols to reduce infectious diseases, especially in young children. Accordingly, the aim of this study was to explore the views of PCPs and parents of young children regarding the practice of hand hygiene in the transmission of diseases in young children.

## Methods

Data for this research were derived from a larger mixed methods qualitative study exploring PCPs and parents’ views, knowledge and attitudes towards their hand hygiene practice and reducing RTI transmission in children < 5 years of age. The methods applied have been previously described [27]; in summary, interviews were conducted with 30 PCPs and five focus groups with 50 parents of young children (see Table 1 for schedules).

**Table 1:** Interview and focus group schedules

PCPs’ semi-structured interview schedule:	
Hand hygiene can reduce respiratory tract infections and gastrointestinal infections:	
a) What do you think of the role of hand hygiene?	
b) What role do you play in promoting hand hygiene?	
c) How do you apply this knowledge to parents/children?	
d) Do you see the need to wash your hands between each child? Even if you don’t make any skin contact with them?	
i) If so, do you wash your hands between each child?	
ii) If not, how often do you wash your hands?	
f) How important do you think hand washing is for respiratory tract infections?	
g) Are there any barriers to hand washing? If so, what are they?	
h) What types of interventions do you think will help you sustain good hand hygiene behaviour? Ie. patient pressure, peer pressure, education, posters	
i) Do you mention hand hygiene as a prevention measure to reducing respiratory infections to parents?	
j) What types of interventions do you think will help parents (and children) to sustain good hand hygiene behaviour?	
Parents’ focus group schedule:	
Hand hygiene can reduce respiratory tract infections and gastrointestinal infections:	
a) What do you think of the role of hand hygiene?	
b) What do you do in terms of hand hygiene at home with your children? Under what circumstances would you remind them to wash their hands?	
c) How important do you think hand washing is for respiratory tract infections?	
d) What barriers or concerns do you have with hand washing?	
e) What types of interventions do you think will help you sustain good hand hygiene habits?	

PCPs were defined as general practitioners (GPs), practice nurses (PNs), maternal child health nurses (MCHNs), and pharmacists (PHs), and a diversified sampling strategy was applied to recruit them. The contact details of GPs and PNs were generated from an existing general practice database at Monash University, Victoria, Australia. Contact details for MCHNs and PHs were generated from the maternal child health services directory [28] and the local business directory respectively. Recruitment was limited to one PCP per practice site across metropolitan Melbourne, Australia.

Purposive sampling via advertisements circulated to playgroups and mothers’ groups was used to target parents and carers from the south east and east of Melbourne, Australia. Five mothers’ groups and play groups were initially approached to recruit the required number of 50 parents and carers. If one site refused due to time or not enough willing participants then another would be approached until the total number of 50 participants were reached. A total of five play groups (two accepted) and three mothers’ group (all three accepted) were approached. Interested participants were asked to contact the researcher (RB). All participants consented to up to an hour interview or focus group to explore their views, knowledge and attitudes towards management of respiratory tract infections, including prevention strategies such as influenza vaccination and hand hygiene in children < 5 years of age.

Interviews and focus groups (each approximately 1 h long) were conducted between June 2014 and July 2015 by RB. PCPs’ were interviewed at their work place or at a place convenient to them during practice hours; focus groups were conducted at play group centres or at scheduled mothers’ group meetings. All participants gave written consent prior to data collection; PCPs were provided with a AUD$120 and parents with a AUD$40 gift voucher upon completion.

Interviews and focus group discussions were digitally recorded and transcribed verbatim. Data were analysed using a thematic approach [29] to provide a flexible approach to identify, analyse and report themes or patterns within the data. Initially, two researchers (RB and BB) read three transcripts independently to generate initial codes and themes, which were then compared and refined until consensus was reached. A further three transcripts were coded using the schemata and this process was repeated, three transcripts at a time, to incorporate emerging themes, until all transcripts were coded. Data were managed using NVivo10. Study approval was obtained from Monash University Human Research Ethics Committee (CF14/1384–2,014,000,648).

## Results

A total of 30 PCPs (13 females) and 50 parents and carers (47 females) participated in the study. The average years of experience for GPs, PNs, MCHNs and PHs were 18.0, 3.0, 17.7, and 7.2 years respectively. In the parents and carers cohort, 62% (*n* = 31) were in the 31–40 years age group, with over 70% (*n* = 36) having a graduate degree or higher.

All participants revealed high levels of knowledge regarding hand hygiene and its importance. When asked, they gave their definition of hand hygiene, and discussed the importance of hand hygiene in reducing transmission of infection, including day to day practice.

*“Washing hands frequently especially after sneezing, touching the nose, touching the mouth, coughing in the hands… the droplets in the transmission and what it means and even touching the handles of the doors, all of these can be a source of infection sometimes, and washing hands, I mean, they are important.” GP11*

*“Yeah I think it's [hand hygiene] quite important, because your hands touch anything. Like your hands will touch the table and someone will come to the table your hands touched - without even realising, you're touching things. Like you're touching your face all day. Scratching your hair, everything, and then you go and touch things…” FG1*

Despite participants having good knowledge of hand hygiene, and recognising the importance in reducing disease transmission, many barriers such as variation in the practice of hand hygiene among PCPs and parents, linking visibility to disease transmission, and doubts that hand hygiene practice was attainable in young children hindered good hand hygiene practice. We elaborate on these themes below.

### Visibility and transmission

Although PCPs unanimously agreed that hand hygiene was important in reducing the transmission of diseases, there were large variations in practice. Three types of hand hygiene practice were identified among GPs and PHs: some would wash hands between seeing patients irrespective of whether contact has been made, some would only wash hands if skin contact was made, while others would practice hand hygiene only if patients were visibly infectious. However, most GPs commented that they would use alcohol sanitizers between patients if hand washing with soap and water was not possible.

*“… every time I examine the patient…” GP11*

*“Not everyone, not if there’s no skin contact…”GP12*

*“…If I’m handling something or I thought they are likely infectious...” GP17*

*“Would be very rare. We don’t try and touch… [we don’t wash hands] not unless they are obviously sick…”PH1*

PNs on the other hand would often wash hands between patients as they were more likely to ‘touch’ patients during procedures, and rarely would MCHNs see babies/children without skin contact. To the latter group, hand hygiene was habitual and ‘routine’.

*“… I regard it [hand hygiene] as a routine…” MCHN1*

Alongside variations in hand hygiene practices among PCPs, there were also divided views about whether to educate parents and patients on hand hygiene during a sick child consultation. Some commented they would if time permitted; some would not as they assumed parents already had good knowledge of hand hygiene and transmission of infection.

*“I do talk to them and tell them it prevents a lot of cross infections…” GP16*

*“…it just doesn’t come up, often there are other things to talk about, and we just don’t have time.” PH4*

*“Look, parents… I don’t know… but I can see most of the parents are quite… they know the hygiene.... They have the knowledge…” GP4*

However, PCPs commented they would not hesitate to discuss hand hygiene during a gastrointestinal tract infection consultation, but they did not always for an RTI consultation. Similar to PCPs, parents also prioritised hand hygiene practice with gastrointestinal infections, which were seen as more infectious as they were more ‘visible’.

*“Just because I think of a cold as being non-severe… Like, just a natural part of life. But gastro just would prefer to avoid.” FG3*

*“Gastro I would [discuss hand hygiene], but not respiratory tract infections.” GP2*

*“But gastro, you’re also vomiting and stuff, and go through places, institutions, like hospitals*…” *GP5*

*“… so when we triage… we do have a chat… like gastro… we have a chat to them about the transmission, and decreasing the spread of virus or whatever is causing the gastro, and what is going around...” PN1*

*“They [pharmacy staff] don’t do it [wash hands] always, but if someone comes in with gastro, they would come straight up and (do the alcohol sanitising motion)…” PH4*

PCPs also commented that the interview process for this study gave them pause for thought making some GPs realise that they need to talk to parents.

While parents considered good hand hygiene as washing hands before meals, after meals and after going to the toilet, similar to PCPs, parents also conflated ‘dirt’ with ‘infectious’ and dirt was a visual cue to prompt them to wash their hands.

*“Just teaching her that if your hands are dirty you wash them, so even though I don’t wash my hands every time I eat, I don’t wash my hands if I’ve been out to the washing line, when she comes in [from outside] - “Oh okay, we’ve got to wash our hands now”” FG2*

*“… if somebody has a cold or somebody has gastro or something like that then I’m really freaky about it and I clean everything within an inch of its life. But then other times, we’re, kind of, more relaxed and pretty lazy about it.” FG3*

Visual cues therefore determined behaviour such as when hands should be washed. Gastrointestinal infections were seen as being ‘visible’, therefore considered as more ‘severe’ than RTIs, leading to the perception that disease transmission and infection control were visually based.

### “Hand hygiene in kids…it’s almost not worth bothering…”

Although PCPs demonstrated good knowledge of transmission of RTIs - respiratory route and fomite transmission – they still insisted that hand hygiene practice would not be effective in preventing or reducing RTI transmission.

*“There is no prevention. I would have to stop sending children to crèche, and kinders, and schools because they get an infection … this is a part of life and growing up … It’s not possible [to prevent]” GP10*

PCPs also believed that hand hygiene could not be achieved in young children as they presumed young children would not have hand hygiene awareness and good practice. In addition, prevention would not be achievable as parents and children have constant contact, especially as young children needed comforting when unwell.

*“Yeah, well, probably not so much in the context of colds, kids are little anyway and they are not going to do it. I talk probably more in terms of gastro, we talk a bit about heightened domestic awareness and practice…” GP7*

*“They are going to kiss you, they are going to touch you… and they are going to kiss each other…” GP2*

Similarly, though parents acknowledged the importance of hand hygiene in reducing transmission of diseases, they also expressed reservations about ‘over-surveying’ their children and becoming ‘germophobic’. Over emphasising hand hygiene was perceived as leading to obsessive behaviours and psychological distress:

“… *I’ve actually had to pull it back because she was in there every five minutes… she got really quite OCD (Obsessive Compulsive Disorder) about the whole thing…” FG1*

*“We sound like we're a bit paranoid… my daughter did say to me that I was turning her into a germ-a-phobe…” FG1*

*“I have seen a lot of quite obsessive hand washers at my new workplace.” FG3*

*“I kind of figured I don’t want to be too paranoid because you can’t wipe your hand every two seconds…” FG4*

While parents did not want to be ‘paranoid’ about being too clean and obsessive about hand hygiene, ultimately, they wanted to find that balance between good hand hygiene practice and not being paranoid about diseases. They did describe struggling to determine what was ‘right’, the ‘correct’ hand hygiene practice, and what was considered as being ‘too clean’. Children being too clean was perceived as weakening immunity whereas being ‘dirty’ built immunity:

*“I also wonder about that whole cause [and] effect. Because the people I know who wash their hands obsessively are always sick. And I just can’t decide if they’re always sick because they’re obsessive hand washers or if they’re obsessive hand washers because they’re always sick…” FG3*

*“I worry about using the hand sanitiser too much… I don't know, I always think there’s … almost too clean…” FG4*

*“I know some people that are clean, I don’t know about too clean, but their kids get sick quite easily. I don’t know whether it’s because they’re not getting immune to some dirt or something…” FG5*

*“We sound like we're a bit paranoid, but that's just us I think.” FG1*

Even though barriers exist for both PCPs and parents of young children when it came to good hand hygiene practice, they all agreed that hand hygiene training still needed to be taught early in life.

### “It really stems from the parents…” – teaching hand hygiene

When asked whose responsibility it was to teach hand hygiene practice to young children, PCPs and parents commented that parents should be responsible.

*“… parents seem to talk to their kids about washing their hands…” GP5*

*“No, I haven’t been telling them, no… I thought the mums would do it…” GP8*

*“So basically, it comes from the parents, if they set good examples…” FG4*

The most effective approach to teaching young children good hand hygiene practice was identified by PCPs and parents as role modelling. Role modelling, the concept of washing hands in front of an audience so the behaviour can be imitated, was expressed as a good way to ‘show’ children how and when hands should be washed, allowing the behaviour to be ‘copied’. Hence developing their hand hygiene practice early in life, and eventually leading to sustained hand hygiene behaviour later in life.

*“I’m role modelling, so they can see me washing my hands… the most important thing I do (in the mother’s group sessions)… that’s hand hygiene.” MCHN1*

*“…Having things down at the children’s level, role-modelling” FG2*

This theme highlighted the general consensus that PCPs and parents thought parents should be responsible for their children’s hand hygiene practice, with prompting and role modeling as the most effective way to teach young children to start the good hand hygiene habit early in life.

## Discussion

Results from this study demonstrated the complex reasoning behind why a simple but important task such as hand hygiene is so difficult to consistently implement in everyday life. Far from a benign, dispassionate process, there are inherent emotions invested in undertaking this task. While the World Health Organization ‘My 5 moments for health hygiene’ recommends health-care workers to clean their hands before touching a patient, before clean/aseptic procedures, after body fluid exposure/risk, after touching a patient, and after touching patient surroundings [30], factors such as the PCP’s own habitual hand hygiene behaviour; the expectation that parents themselves have good hand hygiene practice; scepticism that hand hygiene is effective in reducing RTIs or achievable in young children contributed to the large variation seen in PCPs’ recommendations to promote hand hygiene. For PCPs and parents of young children, hand hygiene practices were centered on visual cues such as gastrointestinal infections and ‘dirt’ as being ‘visible’, rather than the transmission of diseases. While coughing and sneezing can be quite ‘visible’, it is often not associated with being ‘dirty’, hence it is less likely to result in a reflexive action resulting in hand washing. The risk that promoting hand hygiene practice could result in paranoia and the effect of being ‘too clean’ were overriding concerns for parents more so than the message itself.

Variations in practice stemmed from personal attitudes, perceived behaviour, control and subjective norms [31], leading to the intent to wash hands. Some PCPs thought parents were knowledgeable in hand hygiene practice and therefore did not feel the need to mention hand hygiene during an RTI consultation. A recent study by Barroso et al. [25] found a counterintuitive inverse relationship between knowledge and hand hygiene behaviour: where medical students reported high hand hygiene behaviour yet had lower knowledge as compared with medical residents, suggesting that factors other than knowledge were important in determining hand hygiene behaviour in this cohort. Furthermore, many PCPs said they would not wash their hands if there was no patient contact and if the patient was not visibly ‘infectious’. Whitby et al. [32], describe how inherent hand hygiene practice drives the community where visibly soiled, sticky, or gritty hands would prompt hand hygiene behaviour. This ‘perceived susceptibility’ or ‘personal risk’ was also described in a study from eight Mediterranean countries [33], where they found health care workers’ hand hygiene compliance was significantly higher after patient contact compared to before patient contact, implying that self-protection was a major driver of hand hygiene performance in this cohort. Our results indicated that while the importance of hand hygiene was undeniable, hand hygiene practice and passing hand hygiene knowledge to parents of young children varied considerably within and across PCP groups. The diverse situations each PCP face in different scenarios such as whether patients were seen as ‘infectious’, or whether they believed parents have the knowledge as to whether they needed to talk to them about hand hygiene were contributing factors to the variations seen in these groups. Parents also relied heavily on visual cues such as ‘dirty’ and ‘infectious’, to determine the need to hand wash, as they did not always remind their children to wash their hands. However, the ‘awareness’ of hand hygiene practice might also explain that hand hygiene was often taken for granted, and not ‘thought about’. Therefore, behaviour change interventions might need to be regular and applied in small incremental steps. Raising awareness of possible personal risk could improve practice and sustainability when it comes to hand hygiene behaviour [34].

Additionally, parents were reluctant to encourage hand hygiene practices in their child for fear their children would be ‘too clean’, and that they needed to be visibly ‘dirty’ or ‘infectious’ to build their own immunity. This belief needs to be directly challenged by PCPs during discussion in an RTI consultation, and further educating parents on good hand hygiene practice should therefore be considered. A more concerning theme that emerged from our study resulting from the discussions emanating from the parents focus groups was parents’ fear of their child developing abnormal behavior such as OCD. Although studies have shown strong links between people with OCD and feelings driving them to engage repeatedly and excessively in behavior such as hand washing [35, 36], there is no evidence suggesting that hand washing ‘triggers’ OCD. These studies found that OCD was characterized by the reduced ability to terminate an action, such as hand washing, rather than a response to a perceive threat i.e. perceived susceptibility or personal risk. Therefore, parents’ fear of excessing hand washing leading to OCD was not valid. However, the fear was enough for parents to be vigilant with children’s hand washing practice, therefore an important area for further research.

Perhaps one of the biggest barriers to good hand hygiene practice in young children was the skepticism displayed by parents and PCPs that good hand hygiene practice was achievable in young children, and almost not worth pursuing. Thus, while the ‘intent’ was there regarding hand hygiene, compliance did not always follow. Even though successful interventions incorporating hand washing in young children have shown to reduce absenteeism due to infection [9], a recent study of childcare centres in the Netherlands [37] found that while hand hygiene opportunities were readily available for children, overall adherence to hand hygiene guidelines was only 31% in participating day care centres, which supports the publicly held view that hand hygiene practice is not achievable in young children. However, participants in the study also believed that hand hygiene behaviour should start early in life. A study in Seoul, Korea [38], conducted in an elementary school setting with Year 6 students, showed parents’ handwashing practice, parent and child bonding, and shared time have a significant correlation with children’s hand hygiene practice. Our study also suggested that both PCPs and parents thought hand hygiene practice should start with good role modelling in the home, with frequent reminders.

Our study was not without limitations. First, the research was conducted in metropolitan Melbourne, and therefore our results may be not generalisable to other areas such as rural or remote sites, or developing countries where there might be reduced access to hand hygiene products and handwashing facilities. Second, PCPs and parents of young children who participated in the study were very interested in this area, potentially introducing selection bias. Third, providing incentives to participants may have led to a possible source of bias, although these incentives are aligned with similar work with estimated earnings and average Australian wage [39, 40].

Currently little is known regarding young children’s hand hygiene practice in the Australian community. Our study has taken the first step in exploring PCPs’ and parents’ attitude, views and practice of hand hygiene practice, thereby identifying barriers to hand hygiene practice for PCPs and parents of young children, which potentially impact hand hygiene habit and behaviour of young children. To overcome some of these barriers to good hand hygiene practice, the following interventions targeting PCPs and parents may help increase awareness of the importance of hand hygiene and encourage effective hand hygiene behaviour: 1) introduce health promotion that will educate and remind the public that diseases are not always ‘visible’ and that whether or not one appears dirty, transmission is still possible; 2) good hand hygiene habits should be taught early in a child’s life to sustain effective hand hygiene behaviour; and 3) the importance of role modelling as a way to develop good hand hygiene habit in young children. In addition, PCPs should at least encourage parents and young children to practice hand hygiene when coming for an RTI consultation, which may reduce the transmission of RTIs, reinforce the message of the importance of hand hygiene compliance and result in healthy hand hygiene practice in young children. Finally, parents should be reassured that effective hand hygiene practice will not lead to abnormal psychological behaviour in their children and that hand washing will not reduce a child’s immunity.

## Conclusions

This study demonstrated that on the surface, both PCPs and parents of young children thought hand hygiene practice was important. However, dissonance emerged in practice because hand hygiene is implicitly tied to beliefs such as washing hands only when ‘dirty’; concerns that children need to build their immunity and are already too clean; and skepticism that hand hygiene can be achieved in young children. PCPs should be made aware that hand hygiene can be part of the habit of washing hands between patients, due to fomite transmission of diseases in practice. Parental education around the importance of hand hygiene, focused on the tangible goals of making hand hygiene a regular habit is paramount in teaching young children to develop good hand hygiene practice early in life. The decision to perform hand hygiene should not be based on ‘dirt’ or relating visibility of infection to transmission of infection. Rather role modelling hand hygiene by parents as well as enforcing hand hygiene early in the child’s life will help with better hand hygiene compliance leading to reduced transmission of infectious diseases.

## Abbreviations

FG: Focus groups
GP: General Practitioner
MCHN: Maternal and child health nurse
OCD: Obsessive compulsive behaviour
PCP: Primary care providers
PH: Pharmacist
PN: Practice nurse
RTIs: Respiratory tract infections
